# A Review of Bioactive Glass/Natural Polymer Composites: State of the Art

**DOI:** 10.3390/ma13235560

**Published:** 2020-12-06

**Authors:** Rachele Sergi, Devis Bellucci, Valeria Cannillo

**Affiliations:** Dipartimento di Ingegneria Enzo Ferrari, Università degli Studi di Modena e Reggio Emilia, Via P. Vivarelli 10, 41125 Modena, Italy; rachele.sergi@unimore.it (R.S.); devis.bellucci@unimore.it (D.B.)

**Keywords:** natural polymers, bioactive glasses, composites, mechanical properties, biological performance

## Abstract

Collagen, gelatin, silk fibroin, hyaluronic acid, chitosan, alginate, and cellulose are biocompatible and non-cytotoxic, being attractive natural polymers for medical devices for both soft and hard tissues. However, such natural polymers have low bioactivity and poor mechanical properties, which limit their applications. To tackle these drawbacks, collagen, gelatin, silk fibroin, hyaluronic acid, chitosan, alginate, and cellulose can be combined with bioactive glass (BG) nanoparticles and microparticles to produce composites. The incorporation of BGs improves the mechanical properties of the final system as well as its bioactivity and regenerative potential. Indeed, several studies have demonstrated that polymer/BG composites may improve angiogenesis, neo-vascularization, cells adhesion, and proliferation. This review presents the state of the art and future perspectives of collagen, gelatin, silk fibroin, hyaluronic acid, chitosan, alginate, and cellulose matrices combined with BG particles to develop composites such as scaffolds, injectable fillers, membranes, hydrogels, and coatings. Emphasis is devoted to the biological potentialities of these hybrid systems, which look rather promising toward a wide spectrum of applications.

## 1. Introduction

Over the past 40 years, life expectancy in industrialized countries has continued to rise thanks to many factors such as healthier nutrition, health care system and public health efforts, medical treatments, and more salubrious lifestyles [1,2]. However, this progress goes hand in hand with an increase of pathologies and work-related accidents such as spinal problems, arthritis, joint dysfunction, traumatic injuries, and lacerations [3,4]. Such diseases imply dramatic suffering for patients and might cause severe and long-term pain, work limitation, and disability. In this context, biomaterials’ science can make an important contribution to medicine, thanks to the possibility to design increasingly advanced prostheses and implants to be used in several clinical applications to correct and improve irregularities and abnormalities (i.e., spinal rods, pacemaker, stent), to assist in recovery from injury (structural, pharmaceutical effects), and to replace body parts that lose function (total hip, heart). Therefore, new biomaterials with greater biological response, biocompatibility, and thermal and mechanical properties have been increasingly studied [5,6].

Biomaterials, which should be biocompatible, intrinsically non-toxic, non-carcinogenic, and non-allergenic, can be classified as first generation, second generation, and third generation according to their response in the host. First-generation biomaterials had the aim to achieve a proper combination of mechanical and biological properties that correspond to those of the replaced tissue. Typically, these biomaterials do not cause or undergo chemical or biological changes in the host. On the contrary, second-generation biomaterials can promote a specific biological response on the biomaterials’ surface. Therefore, second-generation biomaterials can stimulate the formation of new bone by inducing a controlled action and reaction mechanisms in a biological environment [5,7]. On the other hand, a specific response at the molecular level is promoted by third-generation biomaterials [8]. In fact, such devices can be gradually degraded and substituted by living host tissues.

Biomaterials may include ceramics, glasses, metals, and polymers with their specific properties [9,10]. Since polymers are the most versatile class of biomaterials, they have been extensively used in industrial applications such as farming, food sectors, pharmaceutical, and biomedical fields. Polymers are classified into synthetic and natural types: a large number of synthetic polymers are synthesized from petroleum oil through a series of chemical reactions, while natural polymers are extracted from animal waste or plants in nature [11,12,13]. Synthetic polymers can be classified as (i) hydrophobic, non-water absorbing materials (i.e., polyethylene (PE), polypropylene (PP), poly(methylmethacrylate) (PMMA)); (ii) more polar systems (i.e., copoly(lactic-glycolic acid) (PLGA)) (iii) water-swelling materials (i.e., poly (hydroxyethyl methacrylate) (PHEMA)); and (iv) water-soluble materials (i.e., poly (ethylene glycol) (PEG)). On contrary, natural polymers can be broadly categorized as (i) proteins such as collagen, gelatin, and silk fibroin; (ii) polysaccharides such as chitosan, hyaluronic acid, alginate, and cellulose; (iii) and polynucleotides (DNA, RNA) [12,14].

Regardless of whether they are synthetic or natural, small repeating units constitute the long chain molecule of polymers [12,15]. In synthetic polymers, the small units (i.e., monomers) and the polymerization process influence the characteristic of the final system, such as its crystallinity, molecular weight, and melting temperature [16,17].

Some synthetic polymers lose their integrity once implanted in the body; the degradation kinetic varies from a few weeks to a few years depending on the polymers’ chemical composition and on the surrounding physiological environment [15]. Their gradual degradation begins when the surface of the system starts to absorb components such as water, proteins, and ions from the surrounding environment [15]. Of course, the degradation products released from synthetic polymers should be non-toxic for the host organism. However, as this does not always happen, the efforts of many researchers have been focused on natural polymers, which usually degrade into soluble, non-toxic chemical species [11]. These chemical species are recognizable and metabolized by the body [18], being body-friendly species. Furthermore, synthetic polymers have been partially replaced by natural ones because the latter do not lack in cells recognition sequences [19,20], and the stimulation of a chronic immunological reaction is avoided [21]. Furthermore, natural polymers show more similarity to the extracellular matrix (ECM, network of biomacromolecules including glycosaminoglycans, which are polysaccharides and fibrous proteins such as collagen, laminin, elastin, and fibronectin [12]), being readily recognizable by the body compared to the synthetic ones. Such similarity to ECM could be summarized as a suspension of macromolecules that support everything from local tissue growth to the maintenance of an entire organ. In this context, since ECM provides structural support and modulates the activity of growth factors, it represents one of the main footprints for designing biomaterials [22]. Thus, the ideal biomaterial for tissue engineering applications should be able to recreate the dynamic biochemical, structural, and mechanical properties of the naturally occurring ECM.

However, despite these positive aspects, natural polymers show lower stability in terms of physical and mechanical properties compared to synthetic ones [11]. Moreover, natural polymers suffer some limitations due to their solubility and industrially acceptable processability: (i) variation in the final properties of polymer due to their source, (ii) some contamination caused by the presence of microbes, (iii) uncontrolled water uptake, and (iv) an unpredictable degradation route. Furthermore, since most natural polymers are water-soluble, various crosslinking methods to control their structure, water uptake, and degradation in aqueous environment have been developed [23,24].

Generally, natural polymers have been combined with one another to improve workability; in addition, natural polymers are typically combined with ceramics fillers (i.e., ceramics, glass-ceramics, bioactive glasses) to reinforce the structure of the final system and, thus, to produce composites with a better mechanical performance [25,26]. It is known that bioactive glasses (BGs), and especially the “gold standard” 45S5 Bioglass^®^ [27], have been widely used in several clinical applications in regenerative medicine, tissue engineering, and dentistry [27,28,29,30,31] by virtue of the ability of bonding to bone. The bonding ability to bone of BGs is mediated by the formation of bone-like hydroxy carbonate apatite (HCA) on the surface of BGs once in contact with physiological fluids. The structure and chemistry of HCA is similar to natural apatite, which is the mineral phase of bones; thus, the deposition of HCA on the surface of biomaterials is typically considered one of the initial steps that lead to the formation of a stable bond at the implant/tissue interface [32,33]. Indeed, the rapid physiochemical reciprocal influence between the surface of biomaterials and the surrounding environment with the silica layer and HCA layer formation represent the first step to the success of an implant. Additionally, 45S5 Bioglass^®^ is osteoinductive and able to bond to both soft and hard tissues [34]. However, in recent years, many BGs have been developed, especially those containing the so-called therapeutic ions such as strontium and magnesium [35,36,37,38,39,40,41,42,43,44,45,46,47]. The addition of specific and therapeutic ions that are released during bioactive glass degradation [48,49] aims at enhancing specific cellular response in the host, favoring regenerative processes. Special attention has been paid to the development of sodium containing BGs and/or borate bioactive glasses to improve reactivity in terms of bioactivity and biological response. On the other hand, Cu, Zn, and Ag ions have been incorporated to achieve bioactive glasses with antibacterial properties [50,51,52,53,54]. For these reasons, BGs can be added to natural polymers, which behave as a matrix in order to obtain innovative composites (i) with improved bioactive behavior [55], (ii) with the ability to release therapeutic ions which stimulate a specific molecular response, (iii) and/or with the capability to release alkali ions that counteract the acid by-products from polymers degradation. 

**Figure 1:**
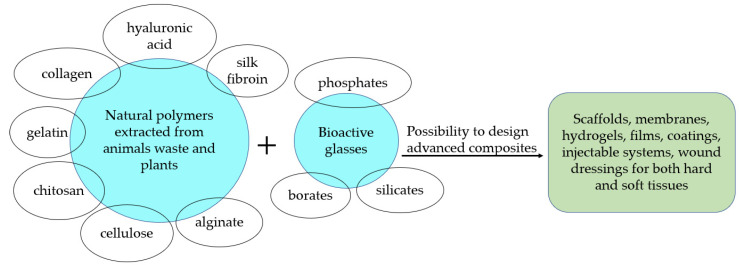
Schematic graph of natural polymers combined with bioactive glasses to fabricate advanced composites.

This review presents the state of the art about the production of composites based on BGs combined with natural polymers: (i) proteins such as collagen, gelatin, silk fibroin, and (ii) polysaccharides such as hyaluronic acid, chitosan, alginate, and cellulose, which are widely used as biomaterials and look particularly promising for several clinical applications. A specific emphasis is given here on the biological performance of such innovative systems, which could open interesting scenarios in the field of biomaterials. Figure 1 schematizes the natural polymers that can be combined with bioactive glasses to fabricate advanced composites, as described in the following.

## 2. Natural Polymers: Proteins

Proteins are assemblies of various amino acids and are monodisperse [12]; various moieties can attach on the amino acid side chain, leading to different physical and biochemical properties of the original protein. Proteins have potential limitations such as immunogenicity as well as batch-to-batch differences consequent to purification processes. Furthermore, proteins materials are generally harvested from animal sources, complicating translation to a clinical setting [56]. Proteins such as collagen, elastin, silk fibroin, and gelatin have been studied as potential scaffolds for tissue applications [12]. Such protein-based materials are useful in sutures, drug delivery vehicles, etc. Proteins-based materials as well as biomaterials in general have to offer tunable degradability providing control over the sequestration and delivery of specific bioactive factors to improve and guide healing and regeneration over a long period. Collagen, gelatin, and silk fibroin with the incorporation of bioactive glasses are considered below.

### 2.1. Collagen/Bioactive Glass Composites

Collagen is a protein that forms the structural basis for much of the extracellular matrix (ECM) of our tissues; therefore, it is the most studied polymer among natural ones.

In the human body, among several types of collagen, types I–IV are the most common [12]. In bone, type I collagen is combined with hydroxycarbonate apatite nanocrystals to create a bio-composite tissue that is highly organized from the nano to macroscopic length scale [57]. In particular, fibers of type I collagen promote cell adhesion and proliferation: cells typically adhere on the collagen surface by integrin α_2_β_1_, which is a primary adhesion receptor used by osteoblast-like cells to bond to the collagen surface (i.e., the substrate), favoring cell–cell interaction and cell–matrix binding [15,58].

Furthermore, collagen stimulates the differentiation of stromal cells of bone marrow [59]. Therefore, as the key component of the ECM, collagen induces bone regeneration and remodeling [60,61] by stimulating bone cells’ metabolic activity [62]. Collagen is suitable for different biomedical applications because it can mimic the mineralization as well as the mechanical and biological properties of osteoid [63]. In addition, its hydrophilic properties, hemostatic capability [12], high processability, and versatility in terms of physical forms give the possibility of using collagen for various products such as injectable gels, films, meshes, and fibers [64,65,66,67], bone and cartilage repair constructs [68,69], skin grafts [70], and wound dressings [71,72].

Despite its versatile form, collagen suffers from a high degradation rate and low mechanical properties (i.e., mechanical strength and stiffness), which can be enhanced by adding a specific reinforcing phase [12,73]. In this context, bioactive glasses (BGs) look particularly interesting as a second phase in a collagen-based composite to improve the mechanical properties of the system as well as the biological ones. It has been reported that bioglass nanofibers added to collagen can reduce infections and induce skin regeneration, thus making the final composite more suitable for clinical uses [73]. Therefore, the appropriate combination of bioactive glasses and collagen leads to biomedical devices that mimic the composition of natural bone. In particular, artificial scaffolds have been developed to solve the limitations of autografts, i.e., limited resources, additional pain, and the morbidity of donor sites [74]. The microporous structure of artificial scaffolds has high porosity and interconnected pores, which permit the spreading and growth of cells, while allowing the flow of nutrients and growth factors (GFs) toward cells and waste products from cells to the surrounding environment [75,76]. This microporous interconnectivity aids cells in multiplying and differentiating into the mature type of tissues [77].

However, an adequate balance between the porosity and bulk structure should be achieved to avoid the failure of structure. In this regard, BGs incorporated in collagen scaffolds resulted in improving compression and elastic modulus compared to pure collagen scaffolds [78]. Furthermore, BGs improved biological responsiveness as well [78]. As mentioned in the introduction, the incorporation of specific ions into the BGs composition enhances the specific cellular response in the host. Indeed, the biological responsiveness and performance of collagen/BGs composites is also associated with bioactive glasses composition [40,79].

The ionic sources such as Ca, Si, and P from bioactive glasses accelerate the precipitation of mineral phase on the surface of scaffolds. The hydroxy carbonate apatite (HCA) was detected on collagen/bioactive glass scaffolds 3 days after soaking in Simulated Body Fluid (SBF) solution [79,80]. The mineralization during immersion in SBF affects the mechanical properties of collagen/45S5 scaffolds, resulting in a transition from a soft to hard tissue with higher resistance to compressive stress [80]. Additionally, the mineralization involves the gradual degradation of BGs, inducing partial degradation of the composite. The degradation rate of composites should be equal to the rate of formation of new tissues [81]. The BGs degradation results in ion releasing, which stimulate the bioactivity as well as cells adhesion and proliferation on collagen/bioactive glass scaffolds compared to pure collagen scaffolds [79,80,82]. Indeed, it is known that the ions released by BGs regulate the gene expression and differentiation of cells to progenitor or stem cells as reported in [83,84]. Once in contact with human body, the typical ions in BGs composition (i.e., Ca, Si, P) influence bone metabolism and angiogenesis. For instance, the Si ion is essential for the metabolic process and for the formation and calcification of bone tissue [85,86]; the Ca ion enhances osteoblast proliferation, differentiation, and extracellular matrix (ECM) differentiation [87,88,89]; the P ion stimulates the expression of matrix protein, which is a key regulation of bone formation [90,91]. Therefore, the introduction of BGs, which release ions directly involved in tissue growth, in particular bone growth, could improve the final properties of collagen/BGs composites compared to collagen scaffolds only [92].

Although scaffolds have acquired extra importance in the regeneration and repair of damaged tissues [93,94,95], to overcome the limit of their rigid structure, injectable systems for small and irregular defects of both hard and soft tissues have been developed [40,82,96,97,98]. Collagen/BGs (CaO-P_2_O_5_-SiO_2_) hydrogels developed by Eglin et al. [96] showed an HCA layer after immersion in SBF solution, confirming that the presence of bioactive glass particles enhances the bioactivity of collagen. The chemical interaction between collagen molecules and bioactive glass nanoparticles should enable a more chemically stable network [99,100], avoiding the faster and total degradation of BGs phase with respect to the degradation of the collagen phase.

Furthermore, the amount of bioactive glass particles into biomedical devices should not exert an excessive inflammatory response during degradation. The inflammatory response could cause an anti-angiogenesis effect, leading to a failure of collagen/BGs composites [97]. For these reasons, the amount of BGs in collagen and the degradation rate of the composite could be controlled by tailoring BGs composition and relative volume fraction, for example. To further control the degradation rate of BG, an improvement of the chemical interaction between collagen and bioactive glass (binary composition of 85% SiO_2_–15% CaO in mol %) should be reached [98]. In this regard, the amination process aims at forming a strong chemical link between the additional amino group of bioactive glasses and the carboxyl group of collagen [98]. This process resulted in a preservation of the original shape and in improving mechanical properties of collagen/85% SiO_2_–15% CaO hydrogels [98].

Improved mechanical properties were measured also for collagen/bioactive glass (BG, Si/Ca/P = 80:15:5) nanofibers developed by the electrospinning technique [101]. Furthermore, collagen/bioactive glass nanofibers promoted the adhesion and proliferation of human dermal fibroblasts (HDFs) cells inducing the secretion of type I collagen (COL-I) and vascular endothelial growth factor (VEGF) [101]. It has been demonstrated that COL-I forms most of the connective tissue in wound healing [102,103] and VEGF stimulates angiogenesis, which could be related to Si ions from BGs, as such ions have been shown to enhance angiogenesis [29,104,105]. The increased angiogenic capability of collagen/bioactive glass composites was coincident with a weaker inflammatory response compared to that of collagen films [101]. By tailoring the amount and composition of bioactive glasses as well as the morphological characteristics of composites, a specific molecular response and improved angiogenesis can be achieved [106,107]. Composites with good mechanical properties and improved biological performances could provide better 3D condition for progenitor or stems cells [108,109,110].

Table 1 summarizes collagen/bioactive glass composites.

### 2.2. Gelatin/Bioactive Glass Composites

Gelatin is a translucent natural nontoxic colorless and brittle natural polymer. It is obtained from collagen that is thermally denaturized or physically/chemically degraded to break its natural triple helix.

The manufacturing process of gelatin consists of (i) the pre-treatment of raw materials for the main extraction step; (ii) the main extraction step, which is the hydrolyzation of collagen into gelatin, and (iii) the final steps to eliminate water from gelatin solution to obtain dried, blended, and ground final gelatin. Being the denaturized form of collagen, gelatin can overcome the concerns about causing an immune response and a transmission of pathogens related to collagen [12].

Furthermore, the unique sequences of amino acids (i.e., glycine, proline, and hydroxyproline) of gelatin influence its biocompatibility and enhance cellular adhesion and proliferation. Therefore, the biocompatibility, biodegradability, and very low antigenicity of gelatin make it appropriate for both hard and soft tissue applications.

Unfortunately, the fast degradation rate in physiological fluids and poor mechanical properties of gelatin limit its application [12]. However, these deficiencies can be mitigated by stabilizing gelatin in a physiological environment using crosslinking agents (i.e., glutaraldehyde and genipin). Since glutaraldehyde could induce cytotoxicity effects, methacrylic anhydride (i.e., a chemical crosslinker) has raised interest due to its inherent biocompatibility, biodegradability, and simplicity in synthesis [111]. Gelatin methacryloyl (GelMA) was produced by adding to type A gelatin (in PBS) the methacrylic anhydride under stirring, as described in detail in [111]. However, gelatin methacryloyl as pure gelatin showed lower ability in promoting tissue regeneration in the absence of bioactive materials. In fact, the development and fabrication of composites for biomedical devices using biodegradable polymers and bioactive materials is currently an open challenge.

In the last decades, bone tissue engineering has become a promising approach to channel this challenge in which composites, bioactive materials, cells, and signaling biomacromolecules could be combined to achieve tissue regeneration.

In this regard, the delivery of cells using carriers (i.e., porous scaffolds) has been shown to be a more efficient method compared to the direct administration of cells in situ. The incorporation of endothelial cells in gelatin/bioactive glass scaffolds further enhanced bone regeneration by improving vascularity compared to simple gelatin/bioactive glass scaffolds [112]. In addition, a novel and high impact gelatin/bioactive glass (64SiO_2_-5P_2_O_5_-31CaO mol%) scaffold, seeded with bone marrow mesenchymal stem cells (BM-MSCs) delivering bone morphogenetic protein-7 (BMP-7), exhibited a successful combination to enhance osteogenesis in bone defects [113]. An appropriate bonding between BM-MSCs/scaffolds and the host bone was related to the capability of bioactive glasses to form bonds with calcified tissues [113].

Indeed, bioactive glasses (BGs) are well known to be both osteoconductive and osteoinductive as well as biocompatible and highly bioactive. Unfortunately, BGs are brittle and exhibit poor mechanical properties, which limits their use in load-bearing applications. Thus, bioactive glasses incorporation into gelatin matrices to fabricate composites represent a feasible solution.

The chemical bonding reaction between the nanoparticles of bioactive glass (nBG, 64SiO_2_-5P_2_O_5_-31CaO mol%) and gelatin is characterized by three major steps as reported elsewhere [114,115,116]: (i) a critical reaction between Ca^2+^ ions of nBG and gelatin molecules; (ii) the Ca^2+^ ions complexing with gelatin molecules, which are assembled with PO_4_^3−^ions; and (iii) the –COOH and –NH_2_ groups in the gelatin molecule form chemical bonds with the P–O and O–H groups of nBG, resulting in a gelatin layer strongly attached to the surface of nBG. 

Gelatin/bioactive glass (64SiO_2_-5P_2_O_5_-31CaO mol%) scaffolds developed for bone tissue applications showed improved mechanical properties compared to gelatin scaffolds and human osteoblast-like cells (SaOS-2) cultured on gelatin/bioactive glass (64SiO_2_-5P_2_O_5_-31CaO mol%) scaffolds were well attached to the pores of scaffolds, as confirmed by their numerous lamellipodia and filopodia [114,115,116]. The SaOS-2 can be used to study mechanisms that involve the late osteoblastic differentiation stage in human cells because of SaOS-2’s capacity to deposit a mineralization-competent extracellular matrix (ECM). In addition, the formation of a hydroxy carbonate apatite layer on the biomaterials’ surface represents one of the necessary requisites to bond with living bone. By incorporating BGs into gelatin matrices, the mineralization was supported compared to pure gelatin composites [116,117,118,119]. The topography, porosity, and surface of scaffolds contributed to a successful interaction with tissues as well as the incorporation of bioactive glasses.

High porous 3D dimensionally interconnected bioactive glass/gelatin composite scaffolds using a direct foaming process were developed [120]. This technique involves little or no organic solvents or hazardous chemicals being an appropriate alternative to conventional techniques used to fabricate synthetic polymer scaffolds and gelatin/BG scaffolds.

Another alternative technique to fabricate scaffolds with a grid-like microstructure was developed by Gao et al. [121]. The solid free form fabrication (SFF) method offered high control on scaffolds structure using a computer-aided design and robotic deposition [121]. Porous 3D gelatin/bioactive glass (70SiO_2_-25CaO-5P_2_O_5_ mol %) scaffolds with high ability to form mineralized bone nodules by combined sol–gel and robocasting technique were developed [121]. On the other hand, the microsphere leaching technique using polymethylmethacrylate (PMMA) as a porogen agent was used to control the porosity of scaffolds [122]. The particle size of the sacrificial polymer spheres determines the porosity; therefore, a well-controlled and open porosity can be obtained by tailoring the size of particles [122]. Furthermore, PMMA can be easily dissolved in acetone, preserving the composite matrix.

During the fabrication of composites containing bioactive glasses, particular attention should be paid to maintaining the bioactive glass composition unchanged. Indeed, the uncontrolled leaching of alkali and alkaline-earth elements alters the property of BGs. The rate of degradation could be partially controlled by tailoring the BGs composition, which is also responsible for a wide range of biological and chemical responses in the host.

In this regard, the incorporation of Sr-doped bioactive glass into gelatin matrix was shown to enhance cell infiltration and neovascularization compared to scaffolds containing bioactive glasses without SrO [123]. The releasing of ions exhibited beneficial effects on bone formation at both cellular and tissue levels [123]. On the other hand, an Mg-Zn doped bioactive glass (SiO_2_-CaO-P_2_O_5_-MgO-ZnO) was added to the gelatin matrix to fabricate composite scaffolds for bone tissue engineering [124], aiming at stimulating cell growth and proliferation. Mg enhances the expression of collagen I and alkaline phosphatase (ALP) activity [37]. Zinc is involved in protein synthesis and is fundamental for the replication of DNA; it is a cofactor for enzymes, and it is engaged during bone cell growth, development, and differentiation [124]. For this reason, ZnO whiskers were added to gelatin/BGs scaffolds to improve cells proliferation; the addition of ZnO whiskers increased rat mesenchymal stem cells (rMSCs) proliferation [125]. Additionally, ZnO whiskers showed antibacterial properties that were favorable for obtaining multifunctional gelatin/BG composite scaffolds with antimicrobial activity [125]. Implanted devices could enhance the chance of infections causing implant failure, hospitalization, and sometime mortality of patients. Therefore, composites with antimicrobial properties would be highly desired. In this regard, a simple and “green” method was used to synthesize in situ silver nanoparticles in gelatin (i.e., a natural reducing and stabilizing agent) by reduction under heating [126]. The incorporation of BGs and silver nanoparticles enhanced human mesenchymal stem cells (hMSC) viability compared to gelatin scaffolds and increased the antibacterial effect of scaffolds with increasing silver nanoparticles [126]. Furthermore, Cu-BGN (95SiO_2_-2.5CaO-2.5CuO mol %)/gelatin coatings were used to successfully coat bioactive glass scaffolds introducing antibacterial properties without compromising the highly interconnected and porous structure of scaffolds [127].

It is worth noting that the concentration of bioactive glass particles in gelatin matrices should be carefully controlled to obtain desired improvements in terms of mechanical and biological properties and antibacterial effects.

Table 2 summarizes gelatin/bioactive glass composites.

### 2.3. Silk Fibroin/Bioactive Glass Composites

Silkworm Bombyx mori (B. mori) and various species of spiders secrete silk fibroin, which is a natural fibrous protein. Since spiders’ silks are different in nature, usually, silks from silkworm are used to fabricate silk-based biomaterials [128]. The raw silk is made of two parallel fibroin fibers held together with a layer of sericin on their surfaces [129]. Sericin should be removed, because it is thought to be responsible for allergic and inflammatory reaction. The fibroin fibers appear shiny and soft to the touch after the degumming process to remove sericin [129]. A light (L) chain polypeptide and heavy (H) chain polypeptide linked together make up the structure of silk fibroin through the formation of the H-L complex [129]. The superior mechanical properties of silk fibroins depend on the H chain, which can form β-sheet crystallites, while the L chain has a small role in the mechanical properties of silk fibroin due to its smaller size [129]. In general, increased β-sheet content has a protective effect on silk degradation, which is likely in part due to the fact that most proteases act outside of the β-sheet regions [130].

Silk fibroin has been widely used for its advantageous strength, biodegradability, elasticity, and low tissue reactivity [12]. In fact, silk fibroin has attractive mechanical properties showing a balance of modulus, breaking strength, and elongation. The hierarchical structure of silk as well as the health of the silkworm (from which silk fibroin is obtained) influence the mechanical properties of silk fibroin [129]. For these favorable properties, silk fibroin has been used as a biomaterial to produce scaffolds, sponges, hydrogels, films, and mats for tissue engineering applications (i.e., vascular tissue, neural tissue, bone tissue [131], cartilage, ligament and tendon tissues [132], wound dressing [133], hepatic tissue, tracheal tissue, etc.).

Although it has these impressive properties, silk fibroin lacks the potential to induce osteogenesis, which is of pivotal importance, especially in bone tissue applications. Therefore, bioactive ceramics (i.e., hydroxyapatite, β-tricalcium phosphates, bioactive glasses) have been added to silk fibroin to fabricate composites that are able to mimic the bone structures.

Among bioactive ceramics, bioactive glasses (BGs) possess greater osteoconductive capacity and a greater ability to bond to bone without forming fibrous tissue. In addition, bioactive glass particles act as reinforcement being beneficial for mechanical properties as well. Indeed, mesoporous bioactive glass (MBG)/silk fibroin scaffolds showed improved compressive strength and better spreading of cells compared to pure silk scaffolds [134] and to non-mesoporous glass/silk scaffolds [135]. Although mesoporous glass destroys the inner structure of silk fibroin, it shows a larger surface area and pore volume with respect to non-mesoporous glass. Thus, during the fabrication process, part of the solution of silk fibroin can enter into the pores of mesoporous glass leading to a stronger bond with silk fibroin after freeze-drying [135]. Additionally, mesoporous glass/silk fibroin scaffolds showed a better apatite mineralization ability probably due to the faster dissolution rate [135,136]. The faster dissolution rate may give a favorable environment for cell adhesion and proliferation and for further tissue growth.

The interaction between biomaterials and cells is known to be a crucial process for the following bone mineralization. Human mesenchymal stem cells (hMSC) cells on silk fibroin hierarchical scaffolding matrices functionalized by a Cu-containing bioactive glass (70S, 70SiO_2_-22CaO-5P_2_O_5_-3CuO molar ratio) coating were found to go toward a differentiated osteoblast phenotype [137]. The Cu-containing bioactive glass coatings improved the ingrowth of new bone tissue in vivo [137].

Ideal scaffolds should possess a structure that mimics the structure of the host tissues in terms of porosity, mechanical, and biological properties to facilitate osteoinduction, vascularization, and tissue formation. In recent years, various strategies to achieve a better control of the micro and macro structural features of scaffolds have been developed ranging from the biphasic structure [138] to an indirect 3D printing technique [139]. The biphasic scaffolds were developed by an electrospinning technique and the two phases (i.e., bioactive glass and silk fibroin) preserved their own porous structure, resulting in good integration at the same time [138]. Therefore, the bioactive glass phase, which has the capability to enhance the osteogenic precursor cells, and the silk fibroin phase, which is able to drive the tissue regeneration, constitute the biphasic scaffolds. In vitro experiments demonstrated an enhanced ALP activity and the absence of adverse immune response [138]. An enhanced ALP activity was also shown by bioactive glass/silk fibroin scaffolds fabricated by an indirect 3D printing technique [139]. The differentiation of osteoblasts was enhanced by the releasing of ions from BGs [139]. Indeed, the incorporation of bioactive glass improved the biological performance of silk fibroin scaffolds, mediating cellular response. The release of ions and surface features such as roughness and hydrophilicity greatly influenced the biological performance. The high hydrophilic nature of bioactive glass/silk fibroin composites compared to pure silk composites enhanced the interaction with cells, which is further improved by BGs, which release ions. Nano bioactive glass particles embedded into silk fibroin films were shown to enhance the hydrophilic nature of silk fibroin, its bioactivity, and ALP activity in in vitro tests [140]. The increase of nano bioactive glass particles in silk fibroin films resulted in more silanol groups on the films surface, which in turn generated an increase in cellular activity. Previous studies have shown that silanol groups bind to proteins through different functional groups, producing a favorable surface environment for cell growth [140].

Table 3 summarizes silk fibroin/bioactive glass composites.

## 3. Natural Polymers: Polysaccharides

Polysaccharides derive from both plant and animal sources and therefore are widely distributed in nature. There are hundreds of known polysaccharides, and they can be categorized based on their type of sources: (i) animals (i.e., hyaluronic acid, chitin, chitosan); (ii) algal (i.e., alginates, galactans, and carrageenan); (iii) plants (i.e., starch, cellulose, arabic gum); and (iv) microorganisms (i.e., dextran, gellan gum, and xanthan gum). The position of O-glycosidic linkages, the molecular weight, and the chain of polysaccharides strongly influence their physical and chemical properties [12]. A considerable number of polysaccharides are water soluble and oxidize beyond their melting point when elevated temperatures are reached. Their tunable degradation and their feasibility make them appropriate for numerous biomedical applications.

### 3.1. Hyaluronic Acid/Bioactive Glass Composites

Hyaluronic acid or hyaluronate is a linear polysaccharide constituted by repeating the disaccharide unit of β(1,4)-glucuronic acid (GlcUA)-β(1,3)-N-acetylglucosamine. Hyaluronic acid is one of the principal parts of extracellular matrix (ECM) [141,142], and it promotes cellular migration and proliferation. The inevitable drawbacks in the extraction process of hyaluronic acid from animal sources are (i) the endogenous hyaluronidase activity in animal tissues and (ii) the difficult conditions of extraction [143]. Additionally, although the hyaluronic acid extracted from animal sources should be extensively purified from contaminants (i.e., proteins and nucleic acids) to reach the high-quality standards for medical applications, the extraction from animal sources remains the principal technology used for commercial products [143], and it is still the most important source for hyaluronic acid. On the other hand, hyaluronic acid can be also synthesized from enzymes; this process still represents a new perspective that can reduce the contamination risks caused by the extraction from animal waste [143].

Hyaluronic acid, along with being used as a component of body lotions, has been used for defective bones and teeth due to its capacity to avoid toxicity, inflammatory, and immunogenicity response in the host. Hyaluronic acid is generally coupled with other polymers (i.e., cohesion promoters) such as chitosan and alginate to improve washout resistance [144,145]. Therefore, hyaluronic acid did not represent the principal polymer used to develop composites [146,147,148,149]. However, hyaluronic acid is selected as a source of polymeric solution because of its excellent biocompatibility, viscoelastic characteristic, and because it is involved in the production of extracellular matrix (ECM) by operating as an organizing core to connect complex intracellular aggregates [150]. Therefore, hyaluronic acid was selected as a polymeric solution by Sohrabi et al. [151] to develop composite injectable pastes with the introduction of bioactive glasses (BGs). The addition of BGs allowed the formation of direct chemical bonds to surrounding tissues, due to their bioactivity and osteoconductivity. Composite injectable pastes must be injected without any resistance and phase separation; the different morphology of bioactive glass embedded into hyaluronic acid influenced the rheological properties of pastes. Bioactive glass particles with high pore volume and low size of particles induce high viscosity of paste. This behavior is probably caused by the penetration of liquids in some pores of BGs reducing available liquid between particles [151]. Although in vitro investigations are surely needed, hyaluronic acid/bioactive glass pastes were developed without coupling polymers [151].

Table 4 summarizes the hyaluronic acid/bioactive glass composite using hyaluronic acid only, without other blending polymers.

### 3.2. Chitosan/Bioactive Glass Composites

Chitosan is a linear polysaccharide formed by a deacetylated unit (β-(1-4)-linked d-glucosamine) and acetylated unit (N-acetyl-d-glucosamine) distributed in a random manner. Chitosan is obtained from shrimps and shells of various crustaceans using alkali NaOH to eliminate the acetamide group (i.e., deacetylation). Deacetylation is never complete: it varies from 30% to 95%.

Chitosan biocompatibility decreases with increasing (i) deacetylation degree (DD), (ii) solubility, and (iii) degradation rate. Additionally, the solubility of chitosan is influenced by the positive charge of amino groups; in fact, chitosan is soluble in diluted acid (pH < 6) only. Furthermore, the hemostatic activity of chitosan is promoted by its positive charge, which is able to bond the negative charge of the membranes of blood cells [152,153]. In fact, chitosan interacts with erythrocyte membranes, attracting platelets and accelerating clot formation [12,153]. The amine and hydroxylic groups enhance the growth of osteoblast and the bone formation in vivo [152,154,155]; the chemical backbone of chitosan is similar to glycosaminoglycan, which is a major component of bones and cartilages. Therefore, chitosan is a suitable candidate for bone grafts [156,157]. Additionally, although the mechanism is still unknown [158,159,160], chitosan shows antimicrobial effects. Two possible mechanisms are considered: (i) the negative charge of chitosan alters the permeability of the membranes of bacteria; and (ii) chitosan prevent the synthesis of RNA by bonding the DNA of bacteria [152,161].

Therefore, chitosan possesses excellent biocompatibility, biodegradability, and antimicrobial effects being a suitable biomaterial for biomedical devices. However, chitosan has low mechanical properties, especially for load-bearing bones [162,163]. To solve this problem by offering synergistic effects among mechanical properties, biocompatibility, and bioactivity, bioactive glasses (BGs) have been widely added to pure chitosan [164]. The improved mechanical properties of chitosan/BG composites were attributed to the addition of BGs, which increased the hardness of chitosan by distributing the applied stress and inhibiting cracks propagation. In general, the addition of BG particles higher than 12.5% w/w led to a significant increase in the storage modulus and specific storage modulus of hydrogels [165]. Presumably, the bonding between the BG surface and chitosan functional groups served to maintain the dispersion of the BG within the chitosan matrix, resulting in better mechanical properties. This hypothesis was corroborated by the storage and loss moduli obtained for bioactive glass-injectable systems, which increased with an increasing amount of BG [166]. Furthermore, the addition of BG nanoparticles also improved the hardness of chitosan fibers as shown in [167]; this enabled also a distribution of the applied stresses over the entire structure of the composite. The compressive strength of chitosan/BG (6Na_2_O, 8K_2_O, 8MgO, 22CaO, 54B_2_O_3_,2P_2_O_5_ mol %) injectable cement well matched the compressive strength of human trabecular and cortical bone [168]. Therefore, chitosan/BG injectable cement could be suitable for healing non-load bearing and load-bearing defects of bone.

Biomaterials are usually employed under hydrated condition (i.e., physiological fluids), which could strongly affect their mechanical properties: such properties are different than the ones under dry condition. In this regard, the mechanical properties of SiO_2_:CaO:P_2_O_5_ = 55:40:5 mol % nanoparticles (BG-NPs) scaffolds were tested under hydrated condition [169], and chitosan/bioactive glass membranes for periodontal regeneration were investigated under both dry and wet conditions [170], since the mechanical properties affect both clinical application and the bone-healing capacity of composites [171,172]. The metabolic activity of cells on chitosan/bioactive glass membranes was higher compared to pure chitosan membranes, indicating a positive effect of bioactive glass incorporation [170]. Therefore, the development of composites aimed at finding a balance between biological performance [173,174,175] and stable structure under mechanical stresses.

The design of the implant’s features, such as the porosity and interconnected pores [176,177] to allow cells migration and nutrients, is relevant as well as ions released from implants. The ionic products of BGs stimulate cell viability and proliferation. For instance, Si ions promoted the production of collagen I and enhanced osteoblastic differentiation [84,105,178,179] and extracellular matrix differentiation [87,89,180]. Additionally, Si ions activated Ca-sensing receptors in osteoblast cells, increasing the expression of insulin-like growth factors (i.e., IGF-I or IGF-II) [49,181,182,183].

Higher cellular viability and a stronger bone formation of chitosan/bioactive glass with high bioactive glass content were measured by Khoshakhlagh et al. [184] compared to that of chitosan/bioactive glass with lower bioactive glass content. By controlling the amount of bioactive glasses inside the chitosan matrix, the degradation rate and the cellular viability could be affected. In addition, by tailoring the chemical composition of bioactive glasses by the addition of different ions (i.e., Sr, Mg, Zn, Ga, Cu), it was possible to stimulate cellular specific responses [178,185]. For instance, Sr ions promoted cellular activity through the membrane-bond Ca-sensing receptor in both osteoblasts and osteoclasts [186,187,188]. Furthermore, Sr ions demonstrated stimulatory effects on osteoblasts, resulting in an increase in bone density and resistance [189,190,191,192]. Indeed, the injectable cement of chitosan and Sr-containing bioactive glasses (CS/SrBG) developed by Zhang et al. [193] significantly enhanced the expression of osteogenic genes and type I collagen, which is an early terminal osteoblast differentiation [192,194]. In vivo CS/SrBG cement enhanced osteogenesis, stimulated bone formation, and showed higher bone–implant contact compared to chitosan/bioactive glass cements without Sr ions. The better ability in improving bone regeneration and bone implant contact was due to the ability of CS/SrBG (i) to mineralize to hydroxy carbonate apatite (HCA), (ii) to promote the delivery of Sr ions in defect areas, and (iii) to improve the secretion of angiogenic factors [193].

On the other hand, Luz et al. [195] developed chitosan/bioactive glass composites with Mg-containing bioactive glasses with enhanced cellular viability and higher ALP activity compared to chitosan/bioactive without Mg ions. As it is well known, Mg ion is not only a cofactor of enzymes that stabilize the structure of RNA and DNA [48,196], but it also influences the phenotype of osteogenic cells and has a pivotal role in the development of skeletal and in the remodeling of bone [107,197]. Furthermore, Mg increases the expression of type X collagen and vascular endothelial growth factor (VEGF), and it increases cell spreading, cell proliferation, and ALP activity [85]. Additionally, chitosan/bioactive glasses with MgO, SrO (BGMS10 [38]), and ZnO (BGMS_2Zn [54]) showed higher cellular viability compared to the cellular viability of chitosan wound dressings [198].

Thus, composites with bioactive glass containing therapeutic ions could represent a promising application for both hard and soft tissues. However, in general, bioactive glasses containing or not some therapeutic ions (i.e., Sr, Mg, Ag, Zn, Cu) are more bio-reactive and show higher capability in stimulating cellular response compared to chitosan matrix. Thus, cells preferentially adhered and proliferated in the area with bioactive glasses with respect to the chitosan area, as found in chitosan/bioactive glass (SiO_2_:CaO:P_2_O_5_ = 55:40:5 mol%) composites developed by microcontact printing [199]. Higher cellular viability and a higher spreading of cells were also found on chitosan/bioactive glass scaffolds developed by Peter et al. [200]. On the contrary, chitosan/bioactive glass (60SiO_2_-36CaO-4P_2_O_5_ mol%) foam developed by Martins et al. [201] did not show higher cell viability compared to pure chitosan foam. However, chitosan/bioactive glass foams showed improved mechanical behavior in terms of elastic modulus and shape recovery compared to chitosan foams. This can be attributed to the chemical crosslinking, which probably resulted from intermolecular reactions between carbonyl and amino groups of chitosan and the silanol group of bioactive glasses [201]. 

Table 5 summarizes chitosan/bioactive glass composites. 

### 3.3. Alginate/Bioactive Glass Composites

Alginate is typically derived from brown algae (Phaeophyceae; including aminaria hyperborean, laminaria digitate, laminaria japonica, ascophyllum nodosum, acrocystis pyrifera) through treatment with aqueous alkali solutions, usually NaOH [202,203,204]. Alginates are formed by (1–4) linked β-D-mannuronic acid (M units) and its C-5 epimer α-L-guluronic acid (G units). The natural copolymer is a component found in algae such as kelp, and an exopolysaccharide found in bacteria (Pseudomonas aeruginosa). M units and G units interspersed with MG sequences form alginates [205].

Even though alginate’s structure is similar to that of an extracellular matrix (ECM), alginate is not degradable inside mammalians because of the absence of the alginate enzyme, which breaks the polymer chain. For this reason, the polymer chain should be partially oxidized with sodium periodate to make alginate degradable in a physiological environment [205]. Therefore, the alginate degradation rate depends especially on the oxidation degree, pH, and temperature of the surrounding environment. Even though the biocompatibility of alginate is still under discussion for possible impurities due to the alginate extraction method [206], alginate is widely used in biomedical applications [204]. Its biocompatibility, biodegradability, hydrophilicity, and relatively low cost make alginate a suitable biomaterial for regenerative medicine [206,207,208]. Additionally, the capability of alginate to generate stable hydrogel in the presence of specific divalent or trivalent cations (i.e., Ca^2+^, Sr^2+^, Al^3+^, Ga^3+^) permits the fabrication of good scaffolds [209].

Alginate hydrogels have been previously used for cell encapsulation [210] and for vascular and cartilage regeneration [211,212]. Additionally, alginate can be dissolved in aqueous solutions without the aid of any solvents. In addition, alginate can be easily modified with peptides, proteins, and inorganic biomaterials. Therefore, alginate is a promising choice for allografts and autografts because alginate does not show the drawbacks such as morbidity and lengthening of the procedure during the process of harvesting [213].

Despite good properties, alginate exhibits poor mechanical strength, bioactivity, and osteoconductivity; thus, bioactive glasses can be combined to ameliorate the biological performance in terms of reactivity [214]. BGs affect the differentiation of osteoblast by increasing the level of ALP, osteocalcin, and osteopontin markers, which control genes involved in cell cycle and progression. Thus, these markers improve osteogenesis by controlling cells during progression toward mature osteoblasts [215]. Additionally, the osteogenesis and ALP activity are directly influenced by the releasing of Si, Ca, Na, and P from the bioactive glass, which activate and upregulate gene expression in cells [216]. In this regard, a higher ALP activity was observed for nano bioactive glass ceramic/alginate scaffolds compared to pure alginate scaffolds [217]. Therefore, the incorporation of bioactive glass contributes to ALP activity, cell adhesion, and proliferation through the release of ions and the increase in the roughness of the composites’ surface [218]. Furthermore, the addition of therapeutic ions could further improve the biological performances of composites, as already mentioned. Thus, 13–93 bioactive glass was used instead of 45S5 to develop composite alginate 3D-printed scaffolds [219]. The addition of 13–93 enhanced the mechanical strength, the mineralization capability of apatite, and the cellular adhesion and differentiation of alginate/13–93 with respect to neat alginate scaffolds. The reason for the higher mechanical strength of alginate/13–93 was that bioactive glass particles showed a high surface area and nanopore volume [220]. The incorporation of 13–93 in alginate increased the pH of PBS, which favored ALP activity; it has been reported that optimum ALP activity occurs at 8.5 pH during bone regeneration [221]. Furthermore, the releasing of Mg^2+^ and SiO^4-^ from 13–93 improved cells’ attachment, proliferation, and differentiation [219]. Indeed, the Mg ion improves cellular proliferation and differentiation, and it has an important role in bone metabolism as well. Mg ions together with Zn ions promoted ALP activity, which stimulated bone metabolism [222]. In addition, the mechanical properties of BGs were improved by the addition of both Mg and Zn ions, because Mg-O and Zn-O bonds have higher bonding energy compared to Ca-O bonds [223].

Therefore, Mg and Zn containing bioactive glass were embedded in alginate to fabricate composite scaffolds with enhanced biological performance and ameliorated mechanical properties [224]. However, the incorporation of bioactive glass in an alginate matrix decreased the micro-porosity and increased the stiffness of alginate. The porosity of scaffolds should guarantee cell spreading and proliferation; thus, the average pore size should be around 100 μm [225]. Even though the porosity decreased with increasing bioactive glass content, a greater cell viability and ALP activity on alginate/bioactive glass (60SiO_2_, 26CaO, 4P_2_O_5_, 5ZnO, and 5MgO mol%) scaffolds compared to alginate scaffolds were detected [224]. This behavior could be due to the dissolution of ions that stimulate the formation of hydroxy carbonate apatite (HCA), which in turns stimulates osteogenic cell attachment and proliferation [226]. In this regard, Sr ions, which promote osteogenic and osteoblast differentiation, are usually added to bioactive glasses [227]. In fact, the Sr ion has been already employed as strontium ranelate in the treatment of osteoporosis due to its anabolic and anti-resorptive effect on bone [228]. Both Sr and Zn act as enhancers in bone formation, simultaneously decreasing bone resorption. The synergistic effect of Sr and Zn rebalances the bone turnover in favor of bone formation, resulting in an increased bone mass and strength [229].

Therefore, Sr and Zn containing bioactive glasses were used as fillers for alginate to improve both mechanical properties and in situ osseointegration [230,231]. The inclusions of bioactive glasses significantly increased the collapse stress/yield stress of the alginate/bioactive glass scaffolds compared to neat alginate scaffolds.

Furthermore, HCA mineralization improves the formation, growth, and maintenance of the tissue–scaffold interface [232,233]. During the immersion, the alkaline pH promotes cells to form new bone [221,234], while the alkaline products of bioactive glasses neutralize the products from alginate degradation [235]. In addition, bioactive glasses decrease the swelling properties of scaffolds because they decrease the polymers surface, which usually binds water molecules [236]. Indeed, the swelling capacity of alginate scaffolds decreased as much as the content of bioactive glasses increased [224,230]. Although it is proved that early swelling enhances cellular adhesion and growth [237,238], a controlled swelling behavior is required for not compromising the mechanical properties of scaffolds [224]. Therefore, to retard and reduce the swelling behavior, alginate is usually crosslinked by bivalent cations such as Ca or Sr, which bond with the carboxyl functional group of G units of the polymer chain [239]. The full crosslinking of alginate limits ions’ availability, allowing control over the kinetics of gels. This is mainly provided by the inclusion of trivalent ions (i.e., Al^3+^ and Ga^3+^), which produce charge balancing and a tetrahedral structure. Ga^3+^ was also crosslinked with alginate in order to control gallium release [240] during degradation. Additionally, Ga/alginate composites showed higher compression strength compared to that measured for Al/alginate composites because Ga is expected to exhibit a higher degree of alginate crosslinking [241]. The compressive strength and elastic modulus increased with an increasing amount of gallium (Ga) in bioactive glass composition as in [242], and the reactive oxygen species (ROS) were reduced by Ga ions [243]. Furthermore, Ga showed an inhibition in bacteria growth [244]: the proposed mechanism is that Ga can disrupt the iron metabolism, increasing the vulnerability of the microorganisms because they are not able to distinguish between Fe^3+^ and Ga^3+^ where the former is redox active and the latter is redox inactive [244]. Therefore, the incorporation of Ga can be used to prevent possible bactericidal colonization of tissue engineering scaffolds after implantation.

On the other hand, a novel combination of glass nanoparticles and alginate to achieve a tunable release capability of Ca^2+^ [245] and Ca^2+^ together with Cu^2+^ were developed [246]. The release of Ca^2+^ and Cu^2+^ enhance cellular growth and differentiation. Such ions stimulated rat bone marrow-derived mesenchymal stem cells (rBMSCs) differentiation toward the osteogenic lineage and improve human umbilical vein endothelial cells (HUVEC) proliferation and vascular endothelial growth factor (VEGF) secretion [246]. Indeed, it is known that Ca^2+^ ions stimulate the differentiation of bone cells, the proliferation of osteoblasts, and the mineralization and metabolism of bone tissue [87,187,188]. On the other hand, Cu^2+^ ions play a pivotal role in bone vessel growth [247,248,249] and in the proliferation of endothelial cells [250,251], being also a natural antimicrobial element [252] showing a synergistic antimicrobial effect with Ca^2+^ [253].

Table 6 summarizes alginate/bioactive glass composites.

### 3.4. Cellulose/Bioactive Glass Composites

Cellulose is the most abundant biopolymer in the biosphere, and it can be obtained from a broad range of plants and animals. Generally, cellulose is obtained from plant sources; however, it can be extracted from bacteria (i.e., bacterial cellulose). The monomer unit of cellulose (β-D-anhydroglucopyranose) is connected covalently through acetal functions to another monomer unit (β-1, 4-glycosidic bonds). These β-1,4-glycosidic bonds guarantee the resistance of cellulose to chemical/enzymatic attack [12,254]. The degree of linearity and –OH groups in the cellulose structure drive the formation of intermolecular and intramolecular hydrogen bonds through the polymer chain [12]. The chains of cellulose are organized in parallel arrangements (i.e., crystalline regions and amorphous-like regions), which influence the physical and chemical properties of cellulose itself [254]. At least five allomorphic forms of cellulose exist: cellulose I, which is the one found in nature, and other crystal structures named cellulose II, III, and IV, where cellulose II is the most stable structure [12]. The three –OH groups in each glucose unit of cellulose influence its reactivity.

Anyways, cellulose has low solubility in common solvents and low dimensional stability, and it shows poor hydrophilicity, bioactivity, and antibacterial properties. Thus, a physical and/or chemical modification of cellulose is required [12].

In addition, bioactive glasses (BGs) can be added to the cellulose matrix to fabricate composites with improved bioactivity and biocompatibility. Cellulose nanofibrils/BG scaffolds with porous microstructure that are able to grow and regenerate bone were developed and investigated [255,256]. By combining cellulose with bioactive glasses, the two main drawbacks (i.e., cellulose bioactivity and bioactive glasses mechanical properties) found in the individual use of such materials could be surpassed. In this sense, the incorporation of CuO oxide, where the Cu ion is known as an essential participant in angiogenesis, could significantly affect the wound healing [255]. The incorporation of CuO did not influence negatively the in vitro mineralization; samples were covered by a hydroxycarbonate apatite (HCA) layer after being soaked in SBF [255,256]. Additionally, the rough surface of HCA favors cell functions in tissue engineering. Such roughness resulted similar to that of cellulose/bioactive glass scaffolds (CC-Bio). These CC-Bio stimulated differentiation into osteoblastic lineage and bone formation [256].

The morphology of the surface in contact with the host tissue is as important as the chemistry of the surface and its capability to release specific ions. For this reason, the surface of metallic devices has been modified with bioactive materials to prevent or postpone second surgeries. The aim was to improve the bone-to-implant contact, reducing infections and surface corrosion.

Cellulose matrix embedding bioactive glasses are considered a promising alternative to conventional bio-ceramic coatings (e.g., [257,258]). The use of cellulose (i) enhances the bond at the interface of surface–devices, (ii) improves the strength of the coatings, (iii) and reduces the releasing rate of ions from bio-ceramic materials. Cellulose nanocrystal (CNC) and bioactive glass coatings on 316L stainless steel by a one-step electrophoretic deposition (EPD) process were fabricated as an alternative to conventional coatings [259,260]. Cellulose acted as a binder, strengthening the connection between the substrate and coatings, leading to a more stable adhesion. The coatings preserved their porous structure as assessed by contact angle measurement [260] and showed higher roughness with respect to the surface of the implant. On the other hand, BGs accelerated the mineralization on the surface of the coatings [259,260] as well as ALP activity, which is the marker of increased bone formation [259].

Table 7 summarizes alginate/bioactive glass composites.

## 4. Additional Remarks

Finally, natural polymers’ advantages and disadvantages, due to their own properties, structure, and methods used to obtain them from natural sources, are summarized in Table 8.

## 5. Conclusions and Future Challenges

The papers reported in this review have shown that collagen, gelatin, silk fibroin, hyaluronic acid, chitosan, alginate, and cellulose containing bioactive glasses represent interesting materials for biomedical devices, for both hard and soft tissue, not least because of their biocompatibility and non-toxicity. The addition of nano-sized and micro-sized bioactive glass particles has been shown to improve mechanical properties, to enhance bioactivity, and to promote the cells’ viability, adhesion, proliferation, and differentiation. However, the strength and cellular response of composites could be compromised when the content of BGs overcome the threshold limit. An appropriate balance between bioactive glasses as filler and good mechanical properties should be considered while developing such composites. Indeed, the hydroxycarbonate apatite on the surface of bioactive glasses has a stimulatory role on the differentiation of stem cells toward osteoblast cells, especially when therapeutic ions are introduced [263]. Additionally, the incorporation of bioactive glasses offers a chance to adjust the elasticity of polymers and the cellular response. The elasticity of natural polymers represents a pivotal feature that affects the cellular response both in vitro and in vivo.

Overall, combining natural polymers with bioactive glass particles to produce composites for both hard and soft tissues can represent an efficient strategy to heal different damages of the body. Considering the in vitro behavior, further research involving the investigation of different cell lines needs to be considered, gaining more relevant data on the osteogenic, odontogenic, and angiogenic behavior of natural polymer composites. Furthermore, it is worth noting that preclinical studies in animals should be improved and become the focus of future studies. In addition, future studies should pay attention to investigate the most advantageous combination of natural polymers and bioactive glasses to achieve good mechanical and biological performances of composites.

Indeed, a better understanding of how specific properties of natural polymer/bioactive glass composites affect cells activation and behavior will allow the optimization of composite production with specific biological responses. Additionally, more investigations on the response induced by specific natural polymers once implanted should be done to further avoid an undesired reaction after implantation.

**Figure 2:**
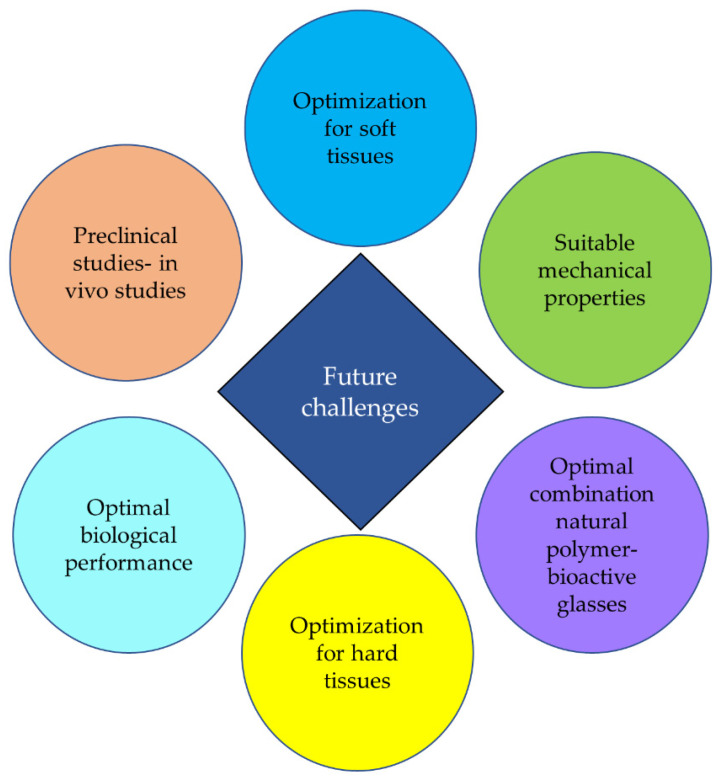
Future challenges.

Figure 2 summarizes future challenges of natural polymer/bioactive glass composites.

## Figures and Tables

**Table 1:** Summary of collagen/bioactive glass composites for various biomedical applications.

Bioactive Glass	Technique	Composites’ Characteristics	Applications	Ref.
BGMS10 (2.3Na_2_O-2.3K_2_O-25.6CaO-10.0MgO-10.0 SrO-2.6 P_2_O_5_-47.2 SiO_2_ mol%)	Manually mixing	Non-cytotoxic; putties containing bioactive glass enhanced cell proliferation	Dentistry and reconstructive surgery	[40]
65SiO_2_-30CaO-5P_2_O_5_ wt%	Slurry-dipping technique	Porosity of 81 ± 4.6% and pore size of 40–200 μm; water adsorption 52.26 ± 11.37%; compression strength 5.80 ± 1.60 MPa; elastic modulus 0.35 ± 0.10 GPa; flexure strength 1.90 ± 0.15 MPa; flexure modulus 27.90 ± 1.32 MPa; tensile strength 3.56 ± 0.22 MPa; tensile modulus 82.00 ± 17.40 MPa. The mechanical properties well-matched with the mechanical properties of trabecular bones; good biocompatibility	Scaffolds for hard tissue	[78]
58S, 70S	Freeze-drying and lyophilization technique	Porosity 87–88%; density 0.0015–0.0016 g/cm^3^. Induced bone mineral-like phase, signs of in vitro bone bioactivity	Scaffolds for hard tissue	[79]
45S5 (nanoparticles)	Plastic compression technique	Good cell viability, high ALP activity, crystalline carbonated hydroxyl apatite growth	Scaffolds for potential bone tissue engineering	[80]
58SiO_2_-38CaO-4P_2_O_5_	Freeze-drying technique	Average thickness 181 μm. Active induction of apatite minerals; significantly high ALP levels	Membranes and scaffolds for bone regeneration	[82]
CaO-P_2_O_5_-SiO_2_ or silica particles	Mixing solution	In vitro osteoconductivity properties	Hydrogels for bone repair and tissue engineering	[96]
45S5	Compression molding	Early angiogenic response	Films for tissue engineering and regenerative medicine	[97]
Mesoporous bioactive glass nanoparticles mBGn (85SiO_2_–15CaO mol%)	Solvent casting	Improved chemical stability, reduced hydrolytic and enzymatic degradation, and increased resistance to loading and stiffness. Storage modulus (E’) of collagen (Col) = 75 kPa, E’ of collagen/bioactive glass (Col-mBGn) = 130–160 kPa and E’ Col–mBGn (Aminated) = 150–280 kPa	Hydrogel for hard tissue engineering	[98]
Si/Ca/P = 80:15:5 mol%	Electrospinning	Improved tensile strength, promotion the secretion of type I collagen (COL-I) and vascular endothelial growth factor (VEGF) in human dermal fibroblast (HDFs), in vivo: skin regeneration in the wound area	Skin wound dressing	[101]

**Table 2:** Summary of gelatin/bioactive glass composites for various biomedical applications.

Bioactive Glass	Technique	Composites’ Characteristics	Applications	Ref.
40SiO_2_-45CaO-15P_2_O_5_	Sequential physical and chemical crosslinking (gelation + UV) approach	Average pore size = 90–200 μm. Compressive modulus: “regular” composites hydrogels (0, 2.5, 5 and 10% w/v BG) = 54.1, 51.2, 50.2, 46.0 kPa; “enhanced” composite hydrogels were approximately 4.4-, 3.8-, 3.5-and 3.3-folds greater than those of “regular” composites hydrogels at 0 to 10% w/v BG. High apatite forming ability. Highly biocompatible; enhanced ALP activity	Hydrogels for bone regeneration	[111]
64S	Layer solvent casting combined with freeze-drying and lamination techniques	Porosity = 85%; average pore size = 200–500 μm. No cytotoxic effect on the cell survival and proliferation in vitro; bone regeneration in vivo	Scaffolds for tissue-engineered bone defects	[112]
64SiO_2_-5P_2_O_5_-31CaO mol%	Freeze-drying technique	Total porosity = 80%; micro-sized porous surface structure = 200–500 μm; elastic modulus = 64 ± 1.3 MPa; compressive yield strength = 4.3 ± 0.23 MPa. No cytotoxic effects; good viability of cells in vitro. Woven bone tissue formation in vivo	Scaffolds for bone defects	[113]
nBG (64SiO_2_ -5P_2_O_5_ - 31CaO mol%)	Layer solvent casting combined with freeze-drying and lamination techniques	Pore size = 200–500 μm; elastic modulus = 50–80 MPa. Good cellular migration and osteoconductivity	Scaffolds for tissue engineering	[114]
BaG (64SiO_2_ -5P_2_O_5_ - 31CaO mol%)	Layer solvent casting combined with freeze-drying and lamination techniques	Parallel aligned and inter-connected pores = 200–500 μm; porosity = 70–86%; Young’s modulus = 50–80 MPa. Improved attachment and penetration of cells into the pores	Scaffolds for bone tissue engineering	[115]
BaG (64SiO_2_ -5P_2_O_5_ - 31CaO mol%)	Freeze-drying technique	Pore size = 200–500 μm; porosity = 70–86%. Young’s modulus: 10% BaG = 51 ± 1.8 MPa, 20% BaG = 58 ± 2.1 MPa, 30% BaG = 64 ± 1.3 MPa, 40% BaG = 72 ± 1.7 MPa, 50% BaG = 78 ± 1.2 MPa. Compressive yield strength: 10% BaG = 2.8 ± 0.26 MPa, 20% BaG = 3.7 ± 0.19 MPa, 30% BaG = 4.3 ± 0.23 MPa, 40% BaG = 4.9 ± 0.30 MPa, 50% BaG = 5.6 ± 0.61 MPa. Improved cell culture response	Scaffolds for bone tissue engineering implant	[116]
45S5	Casting technique	Tensile stress = 0.75–2.1 MPa	Films for soft tissue engineering	[117]
70SiO_2_–25CaO–5P_2_O_5_ mol%	Electrospinning technique	Free from bead-like defects; average fiber diameter = 192 ± 8 nm; tensile strength = 4.3 ± 1.2 MPa; elongation to failure = 168 ± 14%. 14 days after seeding, surfaces covered by multicellular layers; improved ALP activity	Hybrid scaffolds for bone regeneration	[118]
BG (75SiO_2_–25CaO wt%)	Sol–gel method	Porosity = 90%; after immersion in physiological fluids: compressive strength of class I (without covalent linkages between organic–inorganic networks) BG-gelatin = 1 kPa at 10% deformation and of class II (with covalent linkages between organic– inorganic networks) BG-gelatin = 108 kPa at 10% deformation; Young’s modulus class II BG-gelatin = 5-166 MPa; yield strength class II BG-gelatin = 0.2–4 MPa	Hybrid scaffolds for bone regenerative medicine and tissue engineering	[119]
70S30C	Modified direct foaming technique	Modal pore size = 160–170 μm; effective stiffness = 5.87 ± 2.22 MPa; maximum compressive strength = 0.32 ± 0.03 MPa. Samples crosslinked: approximate stiffness = 7.19 ± 2.78 MPa, compressive strength = 0.24 ± 0.04 MPa. Rapid formation of apatite in SBF; excellent cell attachment 3 days after seeding	Scaffolds for bone augmentation and clinical applications	[120]
70SiO_2_-25CaO-5P_2_O_5_ mol%	Solid free form fabrication (SFF) method	Large shrinkage after deposition and drying = 33% linear shrinkage in the plane of deposition and 40–50% in thickness; compressive strength = 3.7 ± 0.2 MPa; compressive strength crosslinked = 5.1 ± 0.6 MPa. Enhanced proliferation, ALP activity, and mineralization of osteogenic MC3T3-E1 (line of mouse pre-osteoblastic cells) in vitro	Hybrid scaffolds for bone regeneration	[121]
75SiO_2_–25CaO wt %	Mixing solution and drying	Total porosity = 91 ± 1%; average pore size = 187 ± 6 μm with pores ranging from 105 to 295 μm; average interconnection diameter = 74 ± 4 μm with diameters ranging from 25 to 115 μm	Scaffolds for tissue engineering	[122]
45S5, BG/Sr (SiO_2_-CaO-SrO-P_2_O_5_) Gel = gelatin	Freeze-drying technique	Pore size: Gel-45S5 15% = 215 ± 12 μm; Gel-BG/Sr 5% = 201 ± 15 μm; Gel-BG/Sr 10% = 164 ± 10 μm; Gel-BG/Sr 15% = 154 ± 16 μm. Porosity: Gel-45S5 15% = 95 ± 2.0%; Gel-BG/Sr 5% = 89 ± 3.25 %; Gel-BG/Sr 10% = 85 ± 1.5 %; Gel-BG/Sr 15% = 80 ± 2.8 %. Young Modulus: Gel-45S5 15% = 14.36 ± 1.3 MPa; Gel-BG/Sr 5% = 10.21 ± 2.0 MPa; Gel-BG/Sr 10% = 19.30 ± 0.68 MPa; Gel-BG/Sr 15% = 70.62 ± 1.91 MPa. ALP secretion significantly enhanced in cells on Gel-BG/Sr15%; cell infiltration and migration enhanced for Gel-BG/Sr15% in vivo	Scaffolds for bone tissue engineering	[123]
SiO_2_-CaO-P_2_O_5_-MgO-ZnO	Freeze-drying technique	Pore diameter in the range of 100–500 μm; average Young’s modulus = 28 ± 2 MPa; average yield strength = 4 ± 0.4 MPa. Insignificant reduction in cells proliferation and no severe toxicity	Scaffolds for bone tissue	[124]
BG (60SiO_2_-36CaO-4P_2_O_5_ mol%) mixed 0 wt%, 1 wt%, 2 wt%, 3 wt%, 4 wt% tetrapod-like ZnO whiskers (indicated as BGZ0, BGZ1, BGZ2, BGZ3, BGZ4)	Freeze-drying technique	Pore size = 100–800 μm; porosity = 80–90%; compression strength (MPa): BGZ0 = 2.62 ± 0.23, BGZ1 = 3.11 ± 0.28, BGZ2 = 3.69 ± 0.30, BGZ3 = 3.24 ± 0.12, BGZ4 = 2.97 ± 0.13. Elastic modulus (MPa): BGZ0 = 112.3 ± 15.9, BGZ1 = 154.6 ± 26.7, BGZ2 = 208.6 ± 31.4, BGZ3 = 147.3 ± 12.6, BGZ4 = 119.6 ± 10.8. Flexure strength (MPa): BGZ0 = 7.28 ± 0.82, BGZ1 = 8.54 ± 1.23, BGZ2 = 9.41 ± 0.72, BGZ3 = 8.48 ± 0.83, BGZ4 = 7.51 ± 1.09. Flexural modulus (MPa): BGZ0 = 612.6 ± 89.5, BGZ1 = 711.5 ± 121.5, BGZ2 = 883.4 ± 45.5, BGZ3 = 756.2 ± 80.9, BGZ4 = 666.0 ± 70.7. Increased proliferation of rat mesenchymal stem cells (rMSCs)	Scaffolds for bone repair	[125]
Silver nanoparticles, 63S	Freeze-drying and crosslinking technique	Pore size of gelatin/nanosilver/bioactive glass scaffolds = 350–635 μm. Gelatin/nanosilver scaffolds high water uptake; gel fraction = 70–85%. Improved human mesenchymal stem cells (hMSC) viability. Antibacterial effects against E. coli and S. aureus	Antibacterial scaffolds for bone tissue engineering	[126]
Cu-BGN (95SiO_2_-2.5CaO-2.5CuO mol%)	Dip coating technique	Porosity = 91%; The obtained scaffolds were designated as 5Cu-BGS and 20Cu-BGS according to the ratios (in wt%) of Cu-BGN/gelatin used (5 and 20 wt%). Compressive strength: 0Cu-BGS = 1.3 ± 0.2 MPa, 5Cu-BGS = 1.2 ± 0.2 MPa, 20Cu-BGS = 0.7 ± 0.3 MPa. Good HCA formation; improved mouse preosteoblastic cell lines (MC3T3-E1) proliferation and improved osteogenic activity	Coatings on BG scaffolds for bone regeneration/repair	[127]

**Table 3:** Summary of silk fibroin/bioactive glass composites for various biomedical applications.

Bioactive Glass	Technique	Composites’ Characteristics	Applications	Ref.
MBG (mesoporous bioactive glass)	3D printing	Compressive strength = 19.9 ± 0.6 MPa. Improved ALP activity. Expression of bone morphogenic protein (BMP-2) = 0.22 ± 0.05	Scaffolds for bone tissue engineering	[134]
Non-mesoporous glass BG, mesoporous bioactive glass MBG (Si/Ca/P = 80/15/5 mol%)	Freeze-drying technique	Porosity: MBG/silk = 76 ± 4%; BG/silk = 76 ± 2%. Compressive strength: MBG/silk = 420 kPa, BG/silk = 300 kPa. Compressive modulus: MBG/silk = 0.7 MPa, BG/silk = 0.5 MPa. MBG/silk and BG/silk good bone repair ability	Scaffolds for bone tissue repair	[135]
Mesoporous bioactive glass nanoparticles (MBGNs)	Freeze-drying technique	Pore diameter = 36.2 ± 9.3 μm. Good adhesion and spreading and proliferation of bone marrow stromal cells (BMSCs)	Scaffolds for orthopedic applications	[136]
70S (70SiO_2_-22CaO-5P_2_O_5_-3CuO mol%)	Freeze-drying and lyophilization technique	Average pore size: -silk fibroin scaffolds without reinforcement 175–145 μm; -silk fibroin matrices reinforced with unmodified silk microfibers 112–143 μm; -composite silk fibroin matrices reinforced with functionalized silk microfibers 108–119 μm. Compressive strength: Silk fibroin matrices reinforced with functionalized silk microfibers 91–123 kPa. Favored homing of endothelial cells through C-X-C chemokine receptor type 4 (CXCR4/SDF-1) signaling in vitro. Facilitated the neo-osseous tissue formation in vivo	Scaffolds as resorbable bone grafts	[137]
70S (70SiO_2_-25CaO-5P_2_O_5_ mol%)	Electrospinning technique	Contact angle bioactive glass/silk fibroin (B. mori silk) biphasic: Silk fibroin side = 73.51 ± 1.8°, bioactive glass side = 24.2 ± 2.2°; contact angle bioactive glass/silk fibroin (A. assama) biphasic: Silk fibroin side = 47.14 ± 2.9°, bioactive glass side = 51.23 ± 2.2°. Elastic modulus: bioactive glass/silk fibroin (B. mori silk) = 29.36 ± 1.38 MPa; bioactive glass/silk fibroin (A. assama) = 27.48 ± 3.96 MPa. Elongation at break: bioactive glass/silk fibroin (B. mori silk) = 2.28 ± 0.59%; bioactive glass/silk fibroin (A. assama) = 8.52 ± 1.43%. Enhanced ALP activity	Biphasic scaffolds for osteochondral tissue repair	[138]
45S5	Combination of indirect 3D inkjet printing and freeze-drying methods	Size of macropores = 500–600 μm; pore size = 20–30 μm. Water uptake: silk fibroin (SF) and 45S5 nanoparticles (nBG) while 45S5 microparticles (μBG) SF-nBG = 85.19 ± 1.6%, SF-μBG = 87.14 ± 0.86%; swelling ratios: SF-nBG = 5.79 ± 0.76%, SF-μBG = 6.79 ± 0.5%. Contact angle: SF-nBG = 63.65 ± 0.74°, SF-μBG = 73.9 ± 0.72°. Compressive strength: SF-nBG = 942 ± 81 kPa, SF-μBG = 1210 ± 80 kPa; compressive modulus: SF-nBG = 8660 ± 660 kPa, SF-μBG = 10353 ± 620 kPa. Viability of cell significantly high on SF-nBG; high ALP activity on SF-nBG and SF-μBG	Scaffolds for bone tissue regeneration in high load-bearing applications	[139]
58sNBG (58SiO_2_-23CaO-9P_2_O_5_ wt%)	Solvent casting method	Thickness = 50–200 μm. Water contact angle = 60–67°. Improved hydrophilicity and in vitro bioactivity. Enhanced proliferation of osteoblasts	Films for bone tissue engineering	[140]

**Table 4:** Summary of hyaluronic/bioactive glass composites.

Bioactive Glass	Technique	Composites’ Characteristics	Applications	Ref.
64SiO_2_-31CaO-5P_2_O_5_	Manually mixing	Better apatite formation ability of bioactive glass/hyaluronic acid (BG2–HAc) compared to bioactive glass/hyaluronic acid (BG1–HAc), where BG1 is the bioactive glass developed by a one-step acid-catalyzed process, while two-step acid–base sol–gel processing was used to produce BG2 bioactive glass. BG2/HAc paste had higher viscosity than BG1/HAc paste.	Injectable pastes for the treatment of hard and even soft tissues	[151]

**Table 5:** Summary of chitosan/bioactive glass composites for hard and soft tissue applications.

Bioactive Glass	Technique	Composites’ Characteristics	Applications	Ref.
BG (46.08SiO_2_-22.96Na_2_O-27.18CaO-3.77P_2_O_5_ wt%)	Solvent casting	Water contact angle chitosan/bioactive glass microparticles (CS/μBG) control = 7.45 ± 6.5°, CS/μBG 7d in PBS = 86.4 ± 6.5°; chitosan/bioactive glass nanoparticles (CS/nBG) control = 65.0 ± 6.3°, CS/nBG 7d in PBS = 81.2 ± 14.6°; Young’s Modulus CS/μBG = 17 MPa, Young’s Modulus CS/nBG = 20 MPa. Excellent hydroxyapatite forming ability	Membranes for bone regeneration	[164]
55SiO_2_-40CaO-5P_2_O_5_ mol%	Dispensing the precursor solutions in wettable spots previously patterned onto superhydrophobic surfaces	Storage modulus = 0.03-5 MPa. Enhanced proliferation and spreading of pre-osteoblast cell line with a fibroblast-like phenotype (MC3T3-E1)	Hydrogels for bone tissue engineering	[165]
BG (55SiO_2_-40CaO-5P_2_O_5_ mol%)	Mixing solution	BG nanoparticles: 0% (control), 10%, 20%, 30%, 40% and 50% (wBG/wchitosan) Storage modulus: BG10 = 24.1 Pa, BG20 = 76.2 Pa, BG30 = 65.0 Pa, BG40 = 105.2 Pa, BG50 = 107.2 Pa. Loss modulus: BG10 = 17.0 Pa, BG20 = 19.8 Pa, BG30 = 16.7 Pa, BG40 = 18.2 Pa, BG50 = 17.7 Pa. Gelation point BG10 = 38.3 °C, BG20 = 29.3 °C, BG30 = 37.0 °C, BG40 = 36.9 °C; BG50 = 36.8 °C	Injectable systems for small bone defects	[166]
60SiO_2_-36CaO-4P_2_O_5_ mol%	Needle punching process	Porosity (%) = 77.52 ± 1.67; water absorption (%) = 58.89 ± 7.3; compression strength = 7.68 ± 0.38 MPa; elastic modulus = 0.46 ± 0.02 GPa; flexure strength = 6.0 ± 0.4 MPa, flexure modulus = 102.0 ± 10 MPa; tensile strength = 3.11 ± 0.24 MPa; tensile modulus = 196.0 ± 17.0 MPa; fracture toughness = 0.24 ± 0.02 MPa m^1/2^. Good biocompatibility	Scaffolds for bone tissue engineering	[167]
6Na_2_O-8K_2_O-8MgO-22CaO-54B_2_O_3_-2P_2_O_5_ mol%	Mixing chitosan solution and glass particles	Injectability = 84–97%; compressive strength = 8–32 MPa; density = 2–3 gcm^−3^; disintegration resistance = 90–95%. Enhanced proliferation and ALP activity; in vivo stimulation of new bone formation	Injectable systems for healing bone defects	[168]
BG-NPs (55SiO_2_-40CaO-5P_2_O_5_ mol%), where NPs means nanoparticles	Freeze-drying technique	Maximum swelling reached for 100 vol% of water = 358%; Young’s Modulus = 55 kPa; strain recovery under deformation strain 30% = 10–80 %; shape fixity ratio = 98.2 ± 0.7 and shape recovery ratio = 89.9 ± 2.7 under deformation strain = 30%. Enhanced apatite formation in vitro	Scaffolds for bone tissue engineering	[169]
55SiO_2_-40CaO-5P_2_O_5_ mol%	Solvent casting	Young’s Modulus = 2639 ± 212 MPa (dry), 4.7 ± 0.3 MPa (wet); ultimate tensile strength = 49.6 ± 9.2 MPa (dry), 3.3 ± 0.6 MPa (wet); elongation at break = 2.5 ± 0.6% (dry), 71.6 ± 11.6 % (wet); water uptake = 130 ± 9 %. Enhanced metabolic activity of human periodontal ligament cells (hPDL) and human bone marrow stromal cells (hBMSC)	Membranes for guided tissue regeneration (GTR) and guided bone regeneration (GBR).	[170]
64SiO_2_ -31CaO-5P_2_O_5_ mol%	Mixing solutions	In vitro hydroxyapatite formation and enhanced cell proliferation. In vivo high rate of new bone regeneration	Injectable system for bone substitute	[184]
Sr-BBG (6Na_2_O-8K_2_O-8MgO-9SrO, 22CaO-54B_2_O_3_-2P_2_O_5_ mol%); BBG (6Na_2_O-8K_2_O-8MgO-22CaO-54B_2_O_3_-2P_2_O_5_ mol%)	Manually mixing	Injectability: Sr-BBG = 98 ± 1%, BBG = 95 ± 1%. Compressive strength: Sr-BBG = 19 ± 1 MPa, BBG = 20 ± 1 MPa. Enhanced proliferation and osteogenic differentiation of human bone marrow stromal cells (hBMSCs) in vitro; good capacity to regenerate bone at the implant bone interface in vivo	Injectable system to treat irregularly shaped bone defects	[193]
55SiO_2_-40CaO-5P_2_O_5_ mol%; 64SiO_2_-26CaO-5P_2_O_5_-5MgO mol%	Solvent casting	High hydrophilicity, good osteoblastic response toward cellular differentiation and mineralization	Membranes for guided tissue regeneration: orthopedic field	[195]
45S5, BGMS10 (47.2SiO_2_-2.3Na_2_O-2.3K_2_O-25.6CaO-2.6P_2_O_5_-10MgO-10SrO mol%); BGMS_2Zn (47.2SiO_2_-2.3Na_2_O-2.3K_2_O-25.6CaO-2.6P_2_O_5_-8MgO-10SrO-2ZnO mol%)	Manually mixing	Enhanced bioactivity, cellular viability, and cells migration rate of chitosan/bioactive glass wound dressings	Wound dressings suitable for healing devices	[198]
BG_NPs (55SiO_2_-40CaO-5P_2_O_5_ mol%), where NPs means nanoparticles	Solvent casting	Nucleation and growth of apatite. Density of cells on the patterned substrate increased with increasing culture time	Membranes pattern with BG to promote guided tissue regeneration in the bone side	[199]
Bioactive glass ceramic nanoparticles (nBGCs)	Lyophilization technique	Interconnected pores 150–300 μm, controlled swelling behavior, good attachment and spread of cells	Scaffolds for tissue applications	[200]
60SiO_2_-36CaO-4P_2_O_5_ mol%	Foaming method	Young’s modulus = 750 ± 0.08 KPa; compressive strength = 120 ± 0.09 KPa; toughness = 1936 ± 0.07 KPa. Total porosity = 65.0 ± 3.6%; average pore size = 118.2 ± 8.2 mm; highly interconnected pore system = 99.8 ± 0.1%. Increased swelling at low pH; adequate cells viability	Scaffolds for tissue regeneration and stimulation of healing	[201]

**Table 6:** Summary of alginate/bioactive glass composites for biomedical applications.

Bioactive Glass	Technique	Composites’ Characteristics	Applications	Ref.
Biosilicate^®^	Manual mixing	Injectability time = 198–119 s; storage modulus = 10–77 kPa. Cell viability = 800RFU	Injectable systems for bone regenerative	[209]
80SiO_2_-15CaO-5P_2_O_5_ mol%	Self-crosslinking process	Porosity = 60–75%; compressive strength = 0.3–2.5 MPa; compressive modulus = 5–65 MPa. Relatively excellent apatite formation ability in vitro. Promotion of early cell adhesion	Scaffolds for bone regeneration	[216]
CaO–SiO_2_–P_2_O_5_ nano bioactive glass ceramic particles (nBGC)	Lyophilization technique	Pore size 100–300 μm; swelling ability; limited degradation; enhanced biomineralization. Good protein adsorption; good cell attachment; good cell proliferation	Scaffolds for periodontal tissue regeneration	[217]
Mesoporous bioactive glass (MBG)	3D plotting	Pore size = 190–415 μm; porosity = 50–69%; compressive strength = 0.4–1.6 MPa; compressive modulus = 1.5–6 MPa. Increased apatite mineralization and cytocompatibility	Scaffolds for bone tissue engineering	[218]
13-93 BG	3D printing	Pore size = 250–500 μm; porosity = 65–87%; compressive strength = 9–16 MPa; modulus = 40–80 MPa. Increased apatite mineralization; rat bone mesenchymal stem cells (rBMSC) spread, adhere and improved osteogenical differentiation	-	[219]
60SiO_2_-26CaO-4P_2_O_5_-5ZnO-5MgO mol%	Freeze-drying technique	Pore size = 75–275 μm; compressive strength = 0.2–1.7 MPa; elastic modulus = 3.5–18 MPa; toughness = 0.25–0.75 MJ/m^3^. Improved degradation rate; improved HA formation; good MG-63 cell line (CRL-1427, 14 years, Caucasian, Passage 4) response (viability, attachment and proliferation); restrict growth of both S. aureus and E. coli	Scaffolds for bone tissue engineering	[224]
A0 (36.4SiO_2_-6.0P_2_O_5_-26.5CaO-2.2CaF_2_-26.5SrO-2.2SrF_2_-0Na_2_O-0K_2_O-0ZnO mol%); A1 (44.0SiO_2_-5.0P_2_O_5_-15.0CaO-0CaF_2_ -15.0SrO-0SrF_2_-10.0Na_2_O-10.0K_2_O-1.0ZnO mol%)	Freeze-drying technique	Pore size = 100–133 μm; Young’s Modulus = 1.6–2.8 MPa	Scaffolds for bone applications	[230]
ICIE16M (49.46SiO_2_-6.60Na_2_O-27.27CaO-3.00SrO-6.60K_2_O-3.00MgO-3.00ZnO-1.07P_2_O_5_ mol %); ICIE16 (49.46SiO_2_-6.60Na_2_O-36.27CaO-0SrO-6.60K_2_O-0MgO-0ZnO-1.07P_2_O_5_ mol%)	Freeze-drying technique	Average pore size of 110 µm and maximum pore size of 309 µm; average collapse stress/yield stress = 0.175 ± 0.04 MPa; average Young’s Modulus = 1.83 ± 0.66 MPa	Scaffolds for bone applications	[231]
AL100 (0.33SiO_2_-0.18Al_2_O_3_-0.00Ga_2_O_3_-0.23CaO-0.11P_2_O_5_-0.15CaCl_2_ mol%); AL067 (0.33SiO_2_-0.12Al_2_O_3_-0.06Ga_2_O_3_-0.23CaO-0.11P_2_O_5_-0.15CaCl_2_ mol%); GA067 (0.33SiO_2_-0.06Al_2_O_3_-0.12Ga_2_O_3_-0.23CaO-0.11P_2_O_5_-0.15CaCl_2_ mol%); GA100 (0.33SiO_2_-0.00Al_2_O_3_-0.18Ga_2_O_3_-0.23CaO-0.11P_2_O_5_-0.15CaCl_2_ mol%)	Mixing solutions	Ultimate compressive strength = 10–70 kPa; elastic modulus = 70–340 kPa. High biocompatibility	Hydrogels for applications in tissue enginnering	[241]
46SiO_2_-23Na_2_O-27CaO-4P_2_O_5_ wt%	Solvent casting, drying and crosslinking technique	Young’s modulus = 0.3–1.45 GPa. Improved acellular bioactivity; enhanced osteoblast-like cell proliferation	Films with prophylaxis effect against infections and potential use in bone tissue engineering	[244]
45S5	Solvent casting	Tensile strength = 7 MPa. Improved biomineralization in vitro	Scaffolds for bone tissue engineering	[245]
Nbg: nominal composition close to Bioglass 45S5 (46SiO_2_-27CaO-23Na_2_O-4P_2_O_5_ wt%)	Solvent casting and drying	Ultimate strength = 6.5–7 MPa. Enhanced bioactivity; stimulation of rat bone marrow-derived mesenchymal stem cells (rBMSCs) differentiation; enhanced human umbilical vein endothelial cells (HUVEC) proliferation and vascular endothelial growth factor (VEGF) secretion	Scaffolds for bone tissue engineering	[246]

**Table 7:** Summary of cellulose/bioactive glass composites for biomedical applications.

Bioactive Glass	Technique	Composites’ Characteristics	Applications	Ref.
MBGSi80 (molar ratio Si/Ca/P = 80/15/5); MBGSi78Cu2 (molar ratio Si/Cu/Ca/P = 78/2/15/5); MBGSi75Cu5 (molar ratio Si/Cu/Ca/P = 75/5/15/5	Freeze-drying and lyophilization technique	Angiogenic effect in the angiogenesis assay; enhanced the gene expression	Membranes for wound healing dressings	[255]
BG	Freeze-casting technique	Pore size = 135 ± 33 μm. Improved hydroxyapatite formation; enhanced cellular proliferation	Scaffolds for bone tissue engineering	[256]
45S5	One step electrophoretic co-deposition process	Thickness = 28 μm. Enhanced attachment, proliferation, and differentiation of cells; accelerated mineralization capability	Hybrid coatings for orthopedic implants	[259]
45S5	One-step electrophoretic deposition process	As-deposited thickness = 14.0 ± 0.9 μm; after 14d in SBF thickness = 7.1 ± 0.6 μm	Coatings for bio-functionalization of metallic orthopedic implants	[260]

**Table 8:** Summary of respective advantages and disadvantages of natural polymers previously analyzed.

Polymer	Advantages	Disadvantages	Refs.
Collagen	Biodegradable, biocompatible, hemostatic, easily modifiable and versatile. Compatible with synthetic polymers	Poor mechanical strength and stiffness. Variability of isolated collagen, hydrophilicity	[12,73,261]
Gelatin	Biodegradable, biocompatible, very low antigenicity, good cell recognition	Fast degradation rate in physiological fluids, brittle	[12]
Silk fibroin	Biodegradable, biocompatible, good structural stability, strength, biodegradability, elasticity, low tissue reactivity	Lacks the potential to induce osteogenesis, sericin needs to be removed	[12,128]
Hyaluronic acid	Biodegradable, biocompatible	High risk of biological contamination, high purification costs	[142]
Chitosan	Biodegradable, biocompatible, hemostatic, hydrophilicity, antibacterial activity, its structure allows specific modifications without too many difficulties	Long period for bone formation, crosslinkers needed to maintain the integrity of structure	[262]
Alginate	Biodegradable, non-immunogenicity, hydrophilicity, formation of gels capable of encapsulating cells, drugs and other biological entities, relative low cost	Biocompatibility under discussion for possible impurities due to the alginate extract, partial oxidation required to make alginate degradable in physiological environment	[205,206]
Cellulose	Biodegradable, biocompatible, most abundant	Low dimensional stability, and it showed poor hydrophilicity, bioactivity, and antibacterial properties	[12]
